# Effectiveness of breast cancer screening policies in countries with medium-low incidence rates

**DOI:** 10.11606/S1518-8787.2018052000378

**Published:** 2018-01-29

**Authors:** Qingxia Kong, Susana Mondschein, Ana Pereira

**Affiliations:** I Universidad Adolfo Ibáñez. Escuela de Negocios. Santiago, Chile; II Universidad Adolfo Ibáñez. Escuela de Ingeniería. Santiago, Chile; III Universidad de Chile. Instituto de Nutrición y Tecnología de los Alimentos. Santiago, Chile

**Keywords:** Breast Neoplasms, epidemiology, Early Detection of Cancer, Mammography, Mass Screening, Preventive Health Services, Health Policy

## Abstract

Chile has lower breast cancer incidence rates compared to those in developed countries. Our public health system aims to perform 10 biennial screening mammograms in the age group of 50 to 69 years by 2020. Using a dynamic programming model, we have found the optimal ages to perform 10 screening mammograms that lead to the lowest lifetime death rate and we have evaluated a set of fixed inter-screening interval policies. The optimal ages for the 10 mammograms are 43, 47, 51, 54, 57, 61, 65, 68, 72, and 76 years, and the most effective fixed inter-screening is every four years after the 40 years. Both policies respectively reduce lifetime death rate in 6.4% and 5.7% and the cost of saving one life in 17% and 9.3% compared to the 2020 Chilean policy. Our findings show that two-year inter-screening interval policies are less effective in countries with lower breast cancer incidence; thus we recommend screening policies with a wider age range and larger inter-screening intervals for Chile.

## INTRODUCTION

Breast cancer (BC) is the most common female cancer worldwide^1^. Chile is no exception; new cases of BC have increased steeply, from 19.9 per 100,000 women in 1960 to 34.8 in 2012. In 2009, BC became the first cause of cancer death among women in Chile, with age-adjusted mortality rate of 11.5 per 100,000 women in 2012^1^ ^,^ ^2^.

Many countries have implemented mammography screening programs, with the aim of reducing the BC mortality rate^3^. Randomized controlled trials have shown an overall reduction in mortality in the range of 15% to 30%^4^ ^,^ ^5^. Nonetheless, the cost effectiveness of such efforts – which is particularly salient in settings with limited resources – has been intensely debated^6^.

In Chile, the current policy allocates only two screening mammograms (Mx) to healthy women between the ages of 50 to 54^7^. Because BC is the first cause of female cancer death and its incidence rate has increased in the last decades, the Chilean Ministry of Health plans to provide access to screening Mx every other year for women between the ages of 50 to 69 by 2020^8^ (namely, the 2020 Chilean policy) with a total number of 10 screening Mx. Although the biennial screening program is recommended in most developed countries, such as Belgium, Spain, and the United States (Preventive Services Task Force), there is still discrepancy regarding these guidelines in developed countries^9^ ^,^ ^10^. Because of these conflicting views, it remains obscure whether a biennial policy is desirable for Chile or other countries in a low-resource setting, with a relatively low incidence rate (one third of that in Belgium) and the different medical cost structure compared to those in developed countries^1^ ^,^ ^11^.

We applied a dynamic programming model to evaluate different screening policies using Chilean data such as BC incidence, survival rates, and costs. We aim to find fixed inter-screening interval policies that excel in death rate reduction and cost-effectiveness using 10 Mx and compare them against the 2020 Chilean policy proposed by the government.

## METHODS

### Mathematical Model

We applied a dynamic programming model developed by Kong and Mondschein^12^ to calculate the optimal ages to perform a fixed number of screening Mx in order to minimize the lifetime death rate from BC (software MATLAB R2015a published by MathWorks). The model decides whether or not to perform a screening mammogram every year during a woman's lifetime. The decision balances the immediate benefit of detecting a tumor at an earlier stage (thereby increasing the chances of survival) and the benefit of waiting until the woman is older, when the probability of developing a tumor might be higher. These decisions depend on incidence, false negative rates, and natural progression of the tumor, as a function of age, sensitivity of clinical breast examination, and survival rates at different stages of the tumor. Given an optimal allocation of screening mammograms, the model computes the corresponding expected costs (including the cost of the screening mammograms, direct cost of diagnosis, treatment, follow-up, and false positives), which enables us to perform a cost-effectiveness analysis. The model also offers the flexibility to evaluate the expected lifetime cost and lifetime death rate for any specific screening policy defined by the decision maker.

### Description of Feasible Screening Policies

We focused on fixed inter-screening interval policies that consist of 10 Mx, which is the number considered in the 2020 Chilean policy. A set of feasible policies was defined to perform 10 Mx among women in the age group of 40 to 80 years old. A feasible policy is defined by two parameters: the starting age for the first mammogram and the length of the inter-screening interval (we considered two, three, and four years in this study). We used the notation P: x-y to describe a policy, where x denotes the inter-screening interval and y, the age of the first screening Mx.

We denoted by lowest death rate policy (P:LDR) the optimal policy obtained when solving the dynamic programming model, which guarantees to find the screening policy with the lowest death rate. We remark that this policy does not necessarily lead to a constant inter-screening interval.

We calculated the expected lifetime cost and death rate per 100,000 women for all feasible policies considered in this study. We then built the efficient frontier, which consists of all fixed inter-screening interval policies such that no other policy leads to a lower cost and lower death rate.

### Data Input and Assumptions for the Dynamic Programming Model

*BC incidence rate:* as Chile does not have a National Cancer Registry, we collected the age-specific incidence rate for BC from GLOBOCAN 2012^1^. The GLOBOCAN estimations are based on the national database for mortality and three regional registries (Valdivia, Biobio, and Antofagasta) for age-specific incidence rates for the period 2003–2007.

*Distribution of BC Stages:* according to Prieto^13^, ductal carcinoma in situ (DCIS) accounts for 8.6% of the non-invasive BC, being the most frequent in Chile; stages I to IV account for 18.7%, 42.1%, 23.3%, and 5.3% of the invasive BC, respectively. For ease of estimation of BC progression rate, we combined BC into two stages: early stage, which includes DCIS and BC stages I and II, and advanced stage, which includes BC stages III and IV. Based on estimations of BC natural history and progression by Maillart et al.^14^, we calculated that if a healthy woman develops cancer within a year, the likelihood of the tumor being in early and advanced stage would be 94%, and 6%, respectively.

*BC survival rates by stage*: data was based on survival rates (as of 2009) reported by Serra et al.^15^, which were estimated using 1,485 cases of BC (18–99 years) from a public hospital in Santiago, Chile, during the period 1994–2005. The estimated 10-year survival rate was 85.6% for early-stage BC and 57.7% for advanced-stage BC.

*False positive and negative results*: the estimation was based on data published by Maillart et al.^14^, in which the authors have reported a percentage of false positive results in the range of 4.4%–7.8% and a percentage of false negative results in the range of 15%–25% for early stages and 7%–18% for advanced stages.

*Sensitivity of clinical breast examination*: the data was retrieved from previously published articles^16^ ^–^ ^18^ in which the sensitivity of clinical breast examination has been estimated to be 11% for early stages and 68% for advanced stages.

*BC and overall mortality rate*: the data was extracted from the Chilean National Death Certificate database, which contains birth and death dates and the specific cause of death for every Chilean citizen. The database is highly reliable, with 100% coverage^19^ and more than 99% of death certificates having been completed by a health professional^20^.

*Costs:* we used the direct cost of screening, diagnosis, and treatment reported in 2013 by the *Fondo Nacional de Salud* ^21^ (FONASA), which is responsible for managing the publicly funded Chilean health system. The cost of a screening Mx reimbursed by FONASA was US$28, and the cost associated to a false positive was US$474. Similar to Schousboe^11^, we estimated the cost of treatment for three categories: first-year costs, follow-up costs from the second year onward, and last-year-of-life costs.

### Progress Rate Estimation

A critical input in the mathematical model is the progression rate of the disease from early to advanced stage within one year as a function of age group. Because of the lack of Chilean data, this set of parameters was estimated by best fitting the observed BC mortality rates for the cohort born in 1935–1939. This estimation incorporates features of the progression rates reported in Maillart et al.^14^, – i.e., that cancer is more aggressive at younger ages (25–49 years = 40% within one year), slows down for middle-aged women (50–59 years = 22%), and decreases further for older women (> 60 years = 15%).

### Model Validation

**Figure 1:**
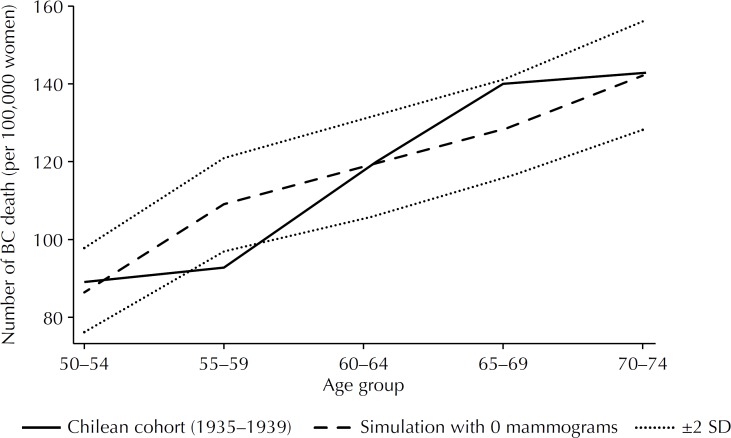
Number of total breast cancer (BC) deaths in 5-year age groups in Chilean women born in 1935–1939 (solid line) and simulation results assuming a population without screening mammography (dashed line).

The dynamic programming model and the input parameter were validated using Chilean data on mortality, and we estimated the BC mortality rate from the cohort of women born in 1935–1939 using national statistics^22^. These women were aged between 60 and 64 years in 2000, and therefore had not participated in the national screening program. Women with more than 12 years of education were excluded, as in general they belonged to a high-income segment and could have undergone opportunistic screening. We then ran extensive simulations of a cohort of 10 million patients with our model, assuming that women had not received any screening Mx. The total age-specific BC deaths were computed with a 95% confidence interval (95%CI) and compared to the actual age-specific BC deaths that occurred in women born in 1935–1939. Figure 1 shows that the mortality of the cohort born in 1935–1939 is included within the 95%CI of the expected mortality rate.

### Cost-effectiveness Analysis

We estimated the average cost of saving an extra life when increasing the number of Mx compared with zero Mx (baseline). The cost-effectiveness ratio (CER) was defined as the additional US dollars spent when doing 10 Mx over the number of lives saved compared to baseline. Additional cost is the difference between the costs of performing 10 Mx and zero Mx, and number of lives saved is the difference between the BC death rate when performing zero Mx and 10 Mx.

$$CER=\frac{Additional\;cost}{Number\;of\;lifes\;saved}=\frac{Total\;cost\;10\;Mx\text{-}Total\;cost\;0\;Mx}{Death\;rate\;0\;Mx\text{-}Death\;rate\;10\;Mx}$$

### Sensitivity Analysis

We performed univariate sensitivity analyses to study the impact of inaccuracy in the estimation of parameters on the structure of the cost-effective screening policies. First, we increased the annual incidence rate in each age group by 10%. Second, because of the increasing trend in the BC incidence rate in Chile^13^, we increased the incidence rate by 1% annually. Finally, we varied false positive and negative values within the range of ±10%.

This research does not require Ethical Institutional Review Board review.

## RESULTS

**Figure 2:**
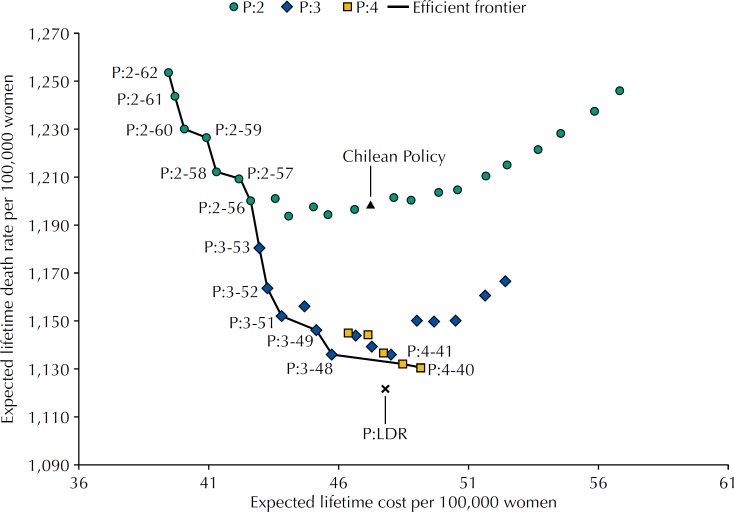
Expected lifetime costs and lifetime death rate per 100,000 women for a set of policies with 10 mammograms and the efficient frontier.

Figure 2 shows the expected lifetime cost and death rate per 100,000 women for different feasible screening policies and the efficient frontier represented by the solid line. The lowest death rate policy P:LDR, obtained by using the dynamic programming model, allocates the 10 Mx at the ages of 43, 47, 51, 54, 57, 61, 65, 68, 72, and 76 and it results on an average of 1,122 deaths per 100,000 women and a CER of US$50,800 per life saved. Among the policies with a fixed inter-screening interval, the two-year inter-screening interval policies are less effective in reducing BC deaths, compared to the three or fouryear inter-screening interval policies. In particular, the 2020 Chilean policy has an average lifetime death rate of 1,199 per 100,000 women and a CER of US$61,100.

Table 1 shows the expected lifetime cost and death rate and the CER for the no-screening policy, the 2020 Chilean policy, and the screening policies belonging to the efficient frontier. The P:LDR reduces the death rate by 26.3% when compared to the no-screening policy (1,522 per 100,000) and by 6.4% when compared to the 2020 Chilean policy. Among the fixed inter-screening interval policies, P:4-40 leads to the lowest death rate (1,131 per 100,000 women) with a CER of $55,500, decreasing the death rate by 5.7% and CER by 9.3%, compared to the 2020 Chilean policy. We also observed that the policy P:3-48 has a slightly higher lifetime death rate (1,136 per 100,000 women) but a considerable lower CER ($47,400), which correspond to a reduction of 5.2% in death rate and 22.5% in CER compared to the 2020 Chilean policy.

**Table 1:** Expected lifetime costs and number of BC deaths per 100,000 women and cost-effectiveness ratio (CER) for 10 mammograms for the no-screening policy, the 2020 Chilean policy, the lowest death rate policy (P:LDR), and the fixed inter-screening interval policies in the efficient frontier.

Policy	Expected cost (US$ millions) per 100,000 women	Expected number of BC deaths per 100,000 women	CER (US$)
No-screening	27.4	1,522	-
2020 Chilean policy (P:2-50)	47.2	1,199	61,142
P:LDR	47.8	1,122	50,847
P:4-40	49.1	1,131	55,472
P:4-41	48.4	1,132	53,869
P:3-48	45.7	1,136	47,355
P:3-49	45.1	1,146	47,080
P:3-51	43.8	1,152	44,191
P:3-52	43.2	1,164	44,088
P:3-53	42.9	1,181	45,436
P:2-56	42.6	1,200	47,203
P:2-57	42.1	1,209	47,065
P:2-58	41.3	1,212	44,749
P:2-59	40.9	1,226	45,473
P:2-60	40.0	1,231	43,341
P:2-61	39.7	1,244	44,030
P:2-62	39.4	1,254	44,697

BC: breast cancer

Table 2 shows the efficient frontier assuming a 10% increase in incidence rate. The efficient frontier is similar to the one obtained in the previous analysis; in particular, P:4-40 still leads to the lowest death rate and, although P:3-48 has a slightly higher death rate, still leads to a lower CER compared to P:4-40. Similar results are observed when considering 1% annual increase in the incidence rate and ±10% variation in the false positive and negative values (data not shown).

**Table 2:** Sensitivity analysis of the expected lifetime costs and number of BC deaths per 100,000 women and cost-effectiveness ratio (CER) for 10 mammograms for the no-screening policy, the 2020 Chilean policy, the lowest death rate policy (P:LDR), and the fixed inter-screening interval policies in the efficient frontier when incidence rate increases by 10% in each age group.

Policy	Expected cost (US$ millions) per 100,000 women	Expected number of BC deaths per 100,000 women	CER (US$)
No-Screening	30.1	1,672	-
2020 Chilean policy	49.9	1,316	55,589
P:LDR	50.4	1,232	46,208
P:4-40	51.8	1,242	50,405
P:4-41	51.1	1,243	48,953
P:3-48	48.4	1,248	43,075
P:3-49	47.8	1,259	42,853
P:3-51	46.5	1,266	40,290
P:3-52	45.9	1,278	40,224
P:3-53	45.7	1,297	41,521
P:2-56	45.3	1,318	43,017
P:2-57	44.8	1,328	42,915
P:2-58	44.0	1,332	40,830
P:2-59	43.6	1,347	41,520
P:2-60	42.8	1,352	39,603
P:2-61	42.4	1,366	40,261
P:2-62	42.2	1,377	40,957

BC: breast cancer

## DISCUSSION

Our results show that the maximum reduction of BC death rate is obtained with the lowest death rate policy (P:LDR), which recommends Mx at ages 43, 47, 51, 54, 57, 61, 65, 68, 72, and 76. However, P:LDR does not have a fixed inter-screening interval, and therefore, it might be difficult to implement it in practice. Among the fixed inter-screening interval policies, P:4-40 leads to the lowest death rate, slightly higher than that in P:LDR (less than 1%). In comparison, P:3-48 has a small increase in death rate (less than 0.5%) but considerable lower CER compared to P4:40. Both policies significantly outperform the 2020 Chilean policy, in terms of death rate and CER. Therefore, decision makers should take these considerations into account when selecting a screening policy that best fits the Chilean population.

Over the years, developed countries have adopted different screening policies, with variations on starting and ending ages and inter-screening intervals^23^. In the last years, these differences have sparked important controversies regarding the effectiveness and efficiency of such policies. For example, in the United States, the National Breast and Cervical Cancer Early Detection Program (NBCCEDP) suggests screening every two years in women aged between 50 and 74 years, providing approximately a total of 13 Mx during their lifetime^24^. This recommendation is similar to some European countries such as France and the Netherlands. In contrast, the United Kingdom offers screening mammograms every three years for women aged between 50 and 69 years, carrying out a total of only seven to eight Mx during a women's lifetime^25^. A recent report commissioned by the Cancer Research UK and the Department of Health of England has estimated that 681 cancer cases will be detected per 10,000 women invited to screening, preventing 43 deaths, but 129 cases will be overdiagnosis cases. The report concluded that, for the case of United Kingdom, it is worth the benefit of carrying out BC screening despite the harms and potential anxiety and suffering for women^10^. We noticed that there is no agreement in the medical community about the effectiveness of screening in the age group of 40 to 49 years^26^. For example, while United Kingdom does not recommend any Mx for women younger than 50, the American Cancer Society recommends annual mammograms for women aged from 45 to 54 years (and every two years after 55) and marks it as optional for women aged from 40 to 44 years^27^. The latter has been thoroughly discussed by Oeffinger et al., with a review of the controlled, observational, and modeling/simulation studies used to support the new recommendations^9^.

We suggest two alternative policies for the Chilean population: P:4-40 and P:3-48, the first one because of its low death rate, and the second one because of its low CER ratio with only a slight increase in death rate. This result is novel because, to the best of our knowledge, none of the existing policies around the world suggest an inter-screening interval of four years and only few countries have adopted a three-year inter-screening interval (e.g. United Kingdom). Our experiments show that, even when the incidence rate increases homogenously among the different age groups, both policies remain desirable. This might result from the complex interaction among limited resources, different cost structure, and inherent patterns in BC incidence and progress rate.

Although the proposed policy P:4-40 and P:3-48 could, on average, have more interval cancers (those not detected during the screening program) compared to those in the 2020 Chilean policy, this increase is counteracted by the cancers detected during the age range that is not covered by the 2020 Chilean policy (40–50 and 68–76). Therefore, a policy with a larger inter-screening interval may not necessarily lead to higher lifetime death rate.

A recent systematic review by Yoo^28^ reports that screening programs are cost-effective in most Western, but not Asian, countries. This could be due to differences in incidence rates and population breast-density distribution. This result agrees with studies suggesting that personalized screening programs based on age, history of BC, breast density, and other known risk factors are more efficient. Schousboe et al.^11^ recommend biannual screening Mx between ages 50 to 79 for women with high-density breasts, and every three to four years for those with low density. Vilaprinyo et al.^29^ recommend different screening frequencies based on risk groups, including annual screening if necessary. In Chile, to classify women into categories according to breast density and to determine screening policies accordingly could be an interesting idea to use scarce resources efficiently. Our mathematical model allows us to determine optimal policies for each risk group based on breast density; however, there are no population studies done in the country to determine the breast density distribution and the quantitative relationship between breast density and incidence rate.

Our study is not free of limitations. When Chilean data was not available, such as false positive/negative percentages, we used available international data. Furthermore, Chile does not have a National Cancer Registry, thus incidence rates were obtained from GLOBOCAN, whose estimations are based on Chilean survival and mortality rates. As shown in the results, the optimal policies do not change when false positive and negative values vary in a range of 10% and incidence rate is modified by 10% in each age group or by 1% annually. In addition, the Chilean cost structure reported by the Ministry of Health is relatively low compared to those in developed countries^11^, and, in particular, the increment in treatment costs from early to advanced stages of cancers is moderate. Therefore, these data suggest that treatment of advanced-stage cancer is still cost-effective. On the other hand, a major strength of our study is the use of an optimization model rather than a simulation one. Thus, we are not only able to evaluate the cost of any current policy, but we can also identify the optimal policy for a given number of screening Mx that minimizes the lifetime mortality rate.

In conclusion, our study indicates that the 2020 Chilean policy is not desirable either in terms of death rate or in terms of cost-effectiveness. Thus, we recommend for Chile or countries with lower BC incidence rate a screening policy that has a wider age range and a larger interscreening interval, compared to most policies implemented in developed countries. In the future, personalized screening could be an option for countries such as Chile that have limited resources and low incidence rate and, therefore, screening efforts could be focused on high-risk population.
